# A Coupled Thermodynamic Model for Transport Properties of Thin Films during Physical Aging

**DOI:** 10.3390/polym11030387

**Published:** 2019-02-26

**Authors:** Hongjiu Hu, Xiaoming Fan, Yaolong He

**Affiliations:** 1 Shanghai Institute of Applied Mathematics and Mechanics, Shanghai University, Shanghai 200072, China; huhongjiu@shu.edu.cn (H.H.); Fan_xm26@163.com (X.F.); 2 Shanghai Key Laboratory of Mechanics in Energy Engineering, Shanghai 200072, China

**Keywords:** physical aging, free volume, transport property, plasticization, carbon dioxide

## Abstract

A coupled diffusion model based on continuum thermodynamics is developed to quantitatively describe the transport properties of glassy thin films during physical aging. The coupled field equations are then embodied and applied to simulate the transport behaviors of O_2_ and CO_2_ within aging polymeric membranes to validate the model and demonstrate the coupling phenomenon, respectively. It is found that due to the introduction of the concentration gradient, the proposed direct calculating method on permeability can produce relatively better consistency with the experimental results for various film thicknesses. In addition, by assuming that the free volume induced by lattice contraction is renewed upon CO_2_ exposure, the experimental permeability of O_2_ within Matrimid^®^ thin film after short-time exposure to CO_2_ is well reproduced in this work. Remarkably, with the help of the validated straightforward permeability calculation method and free volume recovery mechanism, the permeability behavior of CO_2_ is also well elucidated, with the results implying that the transport process of CO_2_ and the variation of free volume are strongly coupled.

## 1. Introduction

The transport property of thin films is one of the most concerning issues for many engineering applications, such as packaging, painting, sensor development, and particularly membrane separation for separating the different components of gaseous streams [1]. Due to the advantages of huge reproducibility for large-scale production and low fabrication cost, as well as high gas selectivity alongside good mechanical performance in comparison to its inorganic and rubbery competitors, polymeric membranes have now dominated membrane separation technology on an industrial scale [2]. However, as amorphous glassy solids, these membranes are in a non-equilibrium state after the materials fabrication, during which they were cooled from above to below their glass transition temperature (*T*_g_). As a result, the glass evolves toward their lowest free-energy state with the passage of time. The process is known as physical aging. This gradual approach to equilibrium affects many properties of amorphous polymers, such as specific volume, permeability, and mechanical response, as well as microstructural or molecular scale properties [3,4,5,6]. Accordingly, how to elucidate the effect of physical aging and thus properly evaluate the behavior of gas separation membranes is essential to ensure satisfactory performance of the films over long service periods for the membrane separation industry.

From a phenomenological perspective, the physical aging of polymers is generally described in terms of the evolution of a non-equilibrium thermodynamic internal variable, for instance, the free volumes of those materials upon their cooling across *T*_g_. Reviews of non-equilibrium behavior and physical aging can be found in the excellent works of Struik [3], Hutchinson [7], and Cangialosi et al. [8]. Upon physical aging, this internal state variable decays spontaneously with time and influences the rate of all time-dependent processes within the polymer. Although some authors [9,10] have suggested that free volume may not be the best parameter to characterize physical aging, the concepts of free volume and free volume distribution are still used widely to describe this aging phenomenon [11,12,13,14,15]. 

Based on the concept of free volume, extensive theoretical and experimental efforts in literatures have been devoted to revealing the aging mechanism of glassy films and to characterize the corresponding permeability degradation. Up to date, the reduction of free volume associated with physical aging aggregates into three mechanisms: (i) Vacancy diffusion, which states the free volume variation as a nonlinear diffusion process; (ii) lattice contraction, which describes the loss of free volume as a self-retarding contraction; and (iii) coupled vacancy diffusion and lattice contraction. Although the above mechanisms have now been widely accepted as the contributing reasons to aging, the quantification on the resulting transport properties still attracts a large number of researchers due to the diversity of experimental results. Among these works, Thornton et al. [16] have coupled the vacancy diffusion model and the Doolittle expression to predict oxygen transport in aging, unaged, and aged poly(vinyl acetate) (PVAc) films. Huang and Paul et al. [17] have analyzed the permeability data of O_2_, N_2_, and CH_4_ within the polysulfone based on bisphenol A (PSF) and Matrimid^®^ 5128 films, as well as poly(2,6-dimethyl-1,4-phenylene oxide) (PPO) films, based on the self-contraction idea. By combining the concepts of lattice contraction and the kinetic theory of glass stabilization proposed by Kovacs [18], Kokou et al. [19] have developed a phenomenological model and simulated the thickness dependence on permeability of He and N_2_ in polynorbornene films ascribed to physical aging. McCaig et al. [20,21] have established a mathematic model based on the coupled diffusion and contraction mechanism to calculate the permeability of O_2_. Their model successfully produces the experimental data for bisphenol-A benzophenone-dicarboxylic acid (BPA-BnzDCA) films with various thicknesses. In later works, Thornton et al. [22] have proposed an empirically derived vacancy diffusion model by a modified form of the Doolittle expression. The advantage of their model is that the free volume can be analytically solved, and it can also predict the experimental permeability of O_2_ by McCaig et al. [20]. Recently, according to the dimensional analysis of the self-contraction mechanism, Müller and Handge et al. [23] have reported a new data processing method and obtained the regression master curves of the tested permeability provided by Rowe et al. [24]. These processed experimental data include the permeability of O_2_, N_2_, and CH_4_ for PSF and Matrimid^®^ films. In the above works, as to associate the free volume $v_{f}$, which characterizes the aging state, with the permeability *P*, a very famous empirical formula proposed by Paul et al. [11] is commonly used, i.e., $P=Ae^{-B/v_{f}}$, where *A* and *B* are constants for a particular gas. 

Although the above studies are significant and highly insightful, and some of them can correlate very well with the experimental values, the evaluation of permeability is indirect and different from the related testing process in which the gas flux across the film is the key for calculating the permeability [25]. Therefore, a definition-based model still needs to be developed. In addition, physical aging is sensitive to the thermodynamic state and the corresponding fraction of free volume within the system. When gas molecules migrate across a glassy polymer film, they would not only occupy part of the free volume space, but also carry energy and/or even plasticize the polymer, which leads to a significant increase of free volume due to the enhanced molecular motions. Consequently, the thermodynamic states of polymer segments and the related aging process of the system can be affected, hinting that the internal transport of mass and the evolution of free volume may be coupled. For example, Paul et al. [26,27,28] have found that when thin (4,4′-hexafluoroisopropylidene) diphthalic anhydride (6FDA) based polyimide membranes were exposed to CO_2_, the permeabilities of all gases would be enhanced, and the effect of CO_2_ can be eliminated though crosslinking, as the thermodynamic performances of the polymer segments have been tailored. In later researches, they have also reported that the tested permeability responses of thin Matrimid^®^ films to experiencing constant CO_2_ exposure for longer periods of time exhibit an initial large increase, followed by a significant decrease in CO_2_ permeability [29]. Although these results are novel, and the interactions between gas transport and aging are implied, the related theoretical work is still lacking.

In this paper, a continuum thermodynamic model which couples the transport of mass and the evolution of free volume will be established. Furthermore, with the purpose of validating the model and demonstrating the underlying coupling phenomenon, the coupled field equations are embodied and applied to the gas separation process. The permeability of gas is specified as an integral function of the concentration gradient, free volume, and free volume gradient by its definition. Hence, the proposed model is validated by the experimental permeability of O_2_ in BPA–BnzDCA films with thicknesses from 0.25 to 33 µm by McCaig et al [21]. Furthermore, by assuming that the free volume induced by lattice contraction is recovered upon CO_2_ exposure, the tested permeability of O_2_ within Matrimid^®^ thin films after a short-time CO_2_ conditioning from the work of Horn and Paul [29] is also reproduced. In addition, the validated simulation method on gas permeability as well as the new proposed free volume recovery mechanism upon CO_2_ exposure are applied further to elucidate the underlying coupling between mass transport and physical aging in Matrimid^®^ thin films with a long-term CO_2_ exposure condition.

## 2. Methodology

Consider a representative element of the polymeric material which undergoes physical aging in gas environments. The mass particles migrate within the polymer due to non-uniform distribution of the chemical potential via available voids, while the available voids, comprising of micro or nano pores and free volumes, change their size or shape in response to the aging of polymeric solids. To mimic the thermo-reversible characteristics of physics within the system, we assume that the transported mass particles do not chemically react with the polymer, and that the evolution of free volume within the element follows the diffusion or contraction mechanisms as described in many physical aging phenomena. Then, following the work of Weitsman et al. [14], the global energy balance and entropy inequality based on the first and second laws of thermodynamics follow:

(1) $$\frac{D}{Dt}\int_{V}\rho_{s_{0}}udV=-\int_{A}q\cdot ndA-\int_{A}\frac{p}{\rho_{c}}f\cdot ndA-\int_{A}u_{c}f\cdot ndA+\int_{A}u_{v}\Psi \cdot ndA+\frac{D}{Dt}\int_{V}u_{v}v_{f}^{LC}dV$$

(2) $$\frac{D}{Dt}\int_{V}\rho_{s_{0}}sdV\geq -\int_{A}\frac{1}{T}q\cdot ndA-\int_{A}s_{c}f\cdot ndA+\int_{A}s_{v}\Psi \cdot ndA+\frac{D}{Dt}\int_{V}s_{v}v_{f}^{LC}dV$$

Here, $\rho_{s}$ is the mass density of the solid, while u and s and are the internal energy and entropy densities of the solid/gas mixture per unit solid mass, respectively. Thus, the items on the left side of Equations (1) and (2) represent the rates of change in energy and entropy within the element, respectively. On the right side, n is the unit vector along the outer normal of the element free surface, while q and *T* are the heat flux and temperature, respectively. The second and third integrals on the right-hand side of Equation (1) are the rate of mechanical work due to gas pressure p and the energy inflow, where $\rho_{c}$, $s_{c}$, and f are the mass density, energy density, and inflow flux, respectively. To describe the thermodynamic state of the aging polymeric material, we assume that physical aging, represented by the variation of free volume, also causes energy and entropy changes with densities of $u_{v}$ and $s_{v}$, respectively. Then, the last two integrals in both Equations (1) and (2) express the rates of change in the internal energy and entropy of the system owing to free volume variation, which comprises of diffusion with flux of Ψ and lattice contraction rate of ${\dot{v}}_{f}^{LC}$.

The global balance of the gas particles as well as the free volume within the element can be given by:

(3) $$\dot{C}=-\nabla \cdot f,\;{\dot{v}}_{f}=\nabla \cdot \Psi +{\dot{v}}_{f}^{LC}$$

where C denotes the concentration of gas and $v_{f}$ is the fractional free volume within the element. The dots above C and $v_{f}$ indicate the time derivative and ∇ represents the gradient operator. Equation (3) tells us that the increase in the rate of gas concentration is driven only by the mass inflow, while the decrease in the rate of fractional free volume in response to the aging process depends on the outflow induced by vacancy diffusion and the lattice contraction rate of ${\dot{v}}_{f}^{LC}$.

Applying Gauss’s theorem to Equations (1) and (2) and taking the Gibbs free energy (G=u−Ts) as the system thermodynamic potential instead of the internal energy u, Equations (1) and (2) can be rewritten as:

(4) $$\rho_{s_{0}}T\frac{ds}{dt}=-\nabla \cdot q-\nabla \cdot \left(hf\right)+\nabla \cdot \left(u_{v}\Psi \right)+u_{v}{\dot{v}}_{f}^{LC}-\rho_{s_{0}}\dot{G}-\rho_{s_{0}}\dot{T}s$$

(5) $$-\rho_{s_{0}}\dot{G}-\rho_{s_{0}}s\dot{T}-\frac{q}{T}\cdot \nabla T-\nabla \cdot \left(\mu_{c}f\right)-s_{c}f\cdot \nabla T+\nabla \cdot \left(R\Psi \right)+s_{v}\Psi \cdot \nabla T+R{\dot{v}}_{f}^{LC}\geq 0$$

where $h=p/\rho_{c}+u_{c}$ and $\mu_{c}=h-s_{c}T$ are the enthalpy and chemical potential of the transported substance, respectively. $R=u_{v}-s_{v}T$ is a thermodynamic potential associated with the free volume, called the free volume potential. 

Assuming further that Gibbs free energy G is a function of mass concentration, temperature, and free volume, i.e., $G=G(C,T,v_{f})$, then the differentiation of G can be expressed as:

(6) $$dG=\frac{1}{\rho_{s_{0}}}\mu_{c}dC-sdT+\frac{1}{\rho_{s_{0}}}Rdv_{f}$$

where $\mu_{c}=\rho_{s_{0}}\partial G/\partial C$,$R=\rho_{s_{0}}\partial G/\partial v_{f}$ and s=−∂G/∂T. 

Application of Equations (3)–(6) yields the differential forms of energy balance and entropy inequality:

(7) $$\rho_{s_{0}}T\frac{ds}{dt}=-\nabla \cdot q-f\cdot \nabla h-s_{c}T\nabla \cdot f+\Psi \cdot \nabla u_{v}+s_{v}T\nabla \cdot \Psi +s_{v}T{\dot{v}}_{f}^{LC}$$

(8) $$\rho_{s_{0}}\dot{s}\geq -\nabla \cdot \left(\frac{q}{T}\right)-\nabla \cdot \left(s_{c}f\right)+\nabla \cdot \left(s_{v}\Psi \right)+s_{v}{\dot{v}}_{f}^{LC}$$

From Equations (7) and (8), the change in entropy consists of flux-related entropy inflow and entropy production, i.e., $ds/dt=-\nabla \cdot s_{1}+s_{2}$, where $s_{1}$ is the flow of entropy and $s_{2}$ is the production of entropy. They provided that:

(9) $$s_{1}=\frac{1}{\rho_{s_{0}}}\left(\frac{q}{T}+s_{c}f-s_{v}\Psi \right)$$

(10) $$\begin{array}{ll} \rho_{s}s_{2} & =-\frac{1}{T^{2}}\nabla T\cdot q-\frac{1}{T}\left(\nabla \mu_{c}+s_{c}\nabla T\right)\cdot f+\frac{1}{T}\left(\nabla R+s_{v}\nabla T\right)\cdot \Psi +s_{v}{\dot{v}}_{f}^{LC}\\ & =\frac{1}{T}(x_{q}\cdot q+x_{f}\cdot f+x_{\Psi }\cdot \Psi )+s_{v}{\dot{v}}_{f}^{LC} \end{array}$$

where

(11) $$\begin{array}{l} x_{q}=-\frac{1}{T}\nabla T\\ x_{f}=-\nabla \mu_{c}-s_{c}\nabla T=-(\frac{\partial \mu_{c}}{\partial T}+s_{c})\nabla T-\frac{\partial \mu_{c}}{\partial C}\nabla C-\frac{\partial \mu_{c}}{\partial v_{f}}\nabla v_{f}\\ x_{\Psi }=\nabla R+s_{v}\nabla T=(\frac{\partial R}{\partial T}+s_{v})\nabla T+\frac{\partial R}{\partial C}\nabla C+\frac{\partial R}{\partial v_{f}}\nabla v_{f} \end{array}$$

are thermodynamic force vectors. Let the fluid flux vector f, heat flux vector q**,** and free volume flux Ψ be expressed in terms of a set of the thermodynamic force vectors $x_{q}$, $x_{f}$, and $x_{\Psi }$ through the phenomenological coefficients $L_{ij}(i,j=1,2,3)$, as stated by Onsager’s principle, i.e.,

(12) $$\begin{array}{l} q=L_{11}x_{q}+L_{12}x_{f}+L_{13}x_{\Psi }\\ f=L_{12}x_{q}+L_{22}x_{f}+L_{23}x_{\Psi }\\ \Psi =L_{13}x_{q}+L_{23}x_{f}+L_{33}x_{\Psi } \end{array}$$

These coefficients $L_{ij}$ may depend on temperature, free volume, and mass concentration [14]. It follows from Equations (11) and (12) that:

(13) $$\begin{array}{l} q=\left(L_{13}(\frac{\partial R}{\partial T}+s_{v})-L_{12}(\frac{\partial \mu_{c}}{\partial T}+s_{c})-L_{11}\frac{1}{T}\right)\nabla T+\left(L_{13}\frac{\partial R}{\partial C}-L_{12}\frac{\partial \mu_{c}}{\partial C}\right)\nabla C+\left(L_{13}\frac{\partial R}{\partial v_{f}}-L_{12}\frac{\partial \mu_{c}}{\partial v_{f}}\right)\nabla v_{f}\\ f=\left(L_{23}(\frac{\partial R}{\partial T}+s_{v})-L_{22}(\frac{\partial \mu_{c}}{\partial T}+s_{c})-L_{12}\frac{1}{T}\right)\nabla T+\left(L_{23}\frac{\partial R}{\partial C}-L_{22}\frac{\partial \mu_{c}}{\partial C}\right)\nabla C+\left(L_{23}\frac{\partial R}{\partial v_{f}}-L_{22}\frac{\partial \mu_{c}}{\partial v_{f}}\right)\nabla v_{f}\\ \Psi =\left(L_{33}(\frac{\partial R}{\partial T}+s_{v})-L_{23}(\frac{\partial \mu_{c}}{\partial T}+s_{c})-L_{13}\frac{1}{T}\right)\nabla T+\left(L_{33}\frac{\partial R}{\partial C}-L_{23}\frac{\partial \mu_{c}}{\partial C}\right)\nabla C+\left(L_{33}\frac{\partial R}{\partial v_{f}}-L_{23}\frac{\partial \mu_{c}}{\partial v_{f}}\right)\nabla v_{f} \end{array}$$

Through substitution of Equation (13) into Equation (3), the governing equations which couple the mass transport process and physical aging in the presence of free volume evolution can be obtained. It should be noted that these formulae only hold at the stress-free state, since the Gibbs free energy is considered to be stress-independent for simplicity, as shown in Equation (6). By introducing additional parameters, such as stress and other internal state variables, more general control equations can be easily obtained via a similar derivation process. These works can refer to Weitsman [14] and Sih et al. [30]. In addition, to determine the evolution of free volume caused by lattice contraction, an additional equation must be introduced. According to the works of Struik [3] and Lock et al. [2], the free volume induced by lattice contraction follows a self-retarding mechanism and can be given by:

(14) $$\frac{d\delta v_{f}^{LC}}{dt}=-\frac{\delta v_{f}^{LC}}{\tau \exp\left(-\gamma \delta v_{f}^{LC}\right)}$$

where $\delta v_{f}^{LC}=v_{f}^{LC}-v_{f_{r}}$ represents the departure of free volume caused by lattice contraction $v_{f}^{LC}$ from its reference equilibrium value $v_{f_{r}}$ and provides the driving force for lattice contraction. τ is the corresponding relaxation time at equilibrium (i.e., at t→∞), while γ is a constant which characterizes the sensitivity of relaxation time to the excess free volume.

## 3. Application to Gas Separation Membranes 

In order to demonstrate the potential coupling between mass transport and physical aging, here we focus on the gas transport properties of the aging separation membranes, which represent one of the most important applications of glassy polymers ascribed to their outstanding separation ability. The thin film is intrinsically one-dimensional that the gas diffusion along the film thickness and is little-affected by stress due to the membrane structure and surface support in service [4], making them a simple and suitable research object. 

For an isothermal physical aging process, the general Gibbs free energy *G* includes three parts, i.e.,

(15) $$G=G_{c}\left(p,C\right)+G_{s}(v_{f})+G_{\mathrm{int}}(p,v_{f})$$

The first part on the right side of Equation (15) is the energy of gas. For an ideal gas in which the interactions between particles are perfectly elastic collisions, it takes the following form:

(16) $$G_{c}\left(p,C\right)=G_{c}\left(p_{0},C_{0}\right)+\frac{kT}{m_{0}}C\ln\left(\frac{p}{p_{0}}\right)$$

where $m_{0}$ is the mass of a single gas molecule and k is Boltzmann’s constant. The second part is the elastic energy in the solid polymer caused by free volume such as vacancy, defects, etc. It is assumed that:

(17) $$G_{s}\left(p,v_{f}\right)=G_{s}\left(p_{0},v_{f0}\right)+\alpha v_{f}^{2}$$

where α is a constant. The third part is the elastic energy caused by gas pressure, which could be small in most cases. Hence, with the help of Equations (15)–(17) and Equations (3) and (13), the coupled partial differential equations taking account of the interaction of the gas diffusion process and physical aging along the film thickness in direction *x* can be given by:

(18) $$\begin{array}{l} \frac{\partial C}{\partial t}=\frac{\partial }{\partial x}\left(L_{22}\frac{\rho_{s_{0}}kT}{m_{0}C}\frac{\partial C}{\partial x}\right)-\frac{\partial }{\partial x}\left(2L_{23}\alpha \rho_{s_{0}}\frac{\partial v_{f}}{\partial x}\right)\\ \frac{\partial v_{f}}{\partial t}=\frac{\partial }{\partial x}\left(2L_{33}\alpha \rho_{s_{0}}\frac{\partial v_{f}}{\partial x}\right)-\frac{\partial }{\partial x}\left(L_{23}\frac{\rho_{s_{0}}kT}{m_{0}C}\frac{\partial C}{\partial x}\right)+\frac{dv_{f}^{LC}}{dt} \end{array}$$

In Equation (18), Onsager’s principle and the nonnegative entropy production assure that the coefficients $L_{22}$, $L_{23}$, and $L_{33}$ are dependent, i.e., $L_{23}\leq \sqrt{L_{22}L_{33}}$. Hence, following the work by Thornton et al. [16], we assume that these coefficients associated with concentration and free volume can be described by a Doolittle expression, i.e.,

(19) $$\begin{array}{l} L_{22}=L_{22}^{0}Ce^{-B_{c}/v_{f}}=\frac{A_{11}m_{0}}{\rho_{s_{0}}kT}Ce^{-B_{c}/v_{f}},\;\;L_{33}=L_{33}^{0}e^{-B_{f}/v_{f}}=\frac{A_{22}}{2\rho_{s_{0}}\alpha }e^{-B_{f}/v_{f}},\\ L_{23}=L_{23}^{0}\sqrt{C}e^{-(B_{c}+B_{f})/2v_{f}}=\frac{A_{12}}{2\rho_{s_{0}}\alpha }\sqrt{C}e^{-(B_{c}+B_{f})/2v_{f}}=\frac{A_{21}m_{0}}{\rho_{s_{0}}kT}\sqrt{C}e^{-(B_{c}+B_{f})/2v_{f}} \end{array}$$

where $A_{11}=\rho_{s_{0}}kTL_{22}^{0}/m_{0}$, $A_{12}=2\rho_{s_{0}}\alpha L_{23}^{0}$, $A_{21}=\rho_{s_{0}}kTL_{23}^{0}/m_{0}$,$A_{22}=2\rho_{s_{0}}\alpha L_{33}^{0}$, and $B_{c}$ and $B_{f}$ are material constants. Therefore, we obtain:

(20) $$\begin{array}{l} \frac{\partial C}{\partial t}=A_{11}\frac{\partial }{\partial x}\left(e^{-B_{c}/v_{f}}\frac{\partial C}{\partial x}\right)-A_{12}\frac{\partial }{\partial x}\left(\sqrt{C}e^{-(B_{c}+B_{f})/2v_{f}}\frac{\partial v_{f}}{\partial x}\right)\\ \frac{\partial v_{f}}{\partial t}=-A_{21}\frac{\partial }{\partial x}\left(\frac{1}{\sqrt{C}}e^{-(B_{c}+B_{f})/2v_{f}}\frac{\partial C}{\partial x}\right)+A_{22}\frac{\partial }{\partial x}\left(e^{-B_{f}/v_{f}}\frac{\partial v_{f}}{\partial x}\right)-\frac{v_{f}^{LC}-v_{f_{r}}}{\tau }e^{\gamma \left(v_{f}^{LC}-v_{f_{r}}\right)} \end{array}$$

From the above equations, we see that the variation of gas concentration is driven by its concentration gradient, and may be affected by the local free volume distribution at the same time. Compared with the concentration gradient term, the negative sign in the last free volume gradient term indicates that the gas molecules have a tendency to migrate from the low free volume region to the high free volume area. Similar interaction effects can also be found in the governing equation of free volume, Equation (23). If the interaction is weak and negligible, i.e., $A_{12}=A_{21}=0$, the two-way coupled equations would degenerate to a decoupled form in which the diffusion of gas molecules in the aging polymer is consistent with the model of Thornton et al. [8], and the governing equations for free volume degenerate into the dual mechanism model proposed by McCaig [21] (γ=0).

For gas separation polymeric membranes, permeability is often used in the industry to characterize the separation productivity of a membrane. It is defined by the flux of penetrant $N_{c}$, the membrane thickness *L*, and the partial pressure or fugacity difference across the membrane Δp, viz. [25],

(21) $$P=N_{c}\cdot L/\Delta p$$

Since the film thickness and pressure difference across the membrane are usually constants in the separation process, the above equation tells us that the change in permeability of an aging membrane is mainly attributed to the variations in flux due to the evolved available free volumes for gas molecules. 

Previously, extensive work has been done to investigate the effect of physical aging on the gas permeability [4,6,11,21,24,28,31,32,33]. Without knowing the flux of the penetrant gas, it has been shown that the permeability is generally determined by correlating to the free volume within the membrane, with $P=Ae^{-B/v_{f}}$. Here, due to coupling between the concentration and free volume, as shown in Equation (20), it is noted that the permeability can also be directly obtained through its definition. With the help of Equation (20), the flux of the penetrant mass is:

(22) $$N_{c}=A_{11}e^{-B_{c}/v_{f}}\frac{\partial C}{\partial x}-A_{12}\sqrt{C}e^{-(B_{c}+B_{f})/2v_{f}}\frac{\partial v_{f}}{\partial x}$$

As the above mass flux is applied point by point, the related permeability from Equation (21) would be a function of *x* and *t*, i.e., P(x,t). To compare with the experimentally observed values of P(t), an average permeability value over the film thickness is therefore applied in the following simulations, i.e.,

(23) $$P\left(t\right)=\frac{{\bar{N}}_{c}\cdot L}{\Delta p}=\frac{1}{\Delta p}\int_{0}^{L}\left(A_{11}e^{-B_{c}/v_{f}}\frac{\partial C}{\partial x}-A_{12}\sqrt{C}e^{-(B_{c}+B_{f})/2v_{f}}\frac{\partial v_{f}}{\partial x}\right)dx$$

## 4. Results and Discussion

### 4.1. Model Validation and Comparison

**Figure 1:**
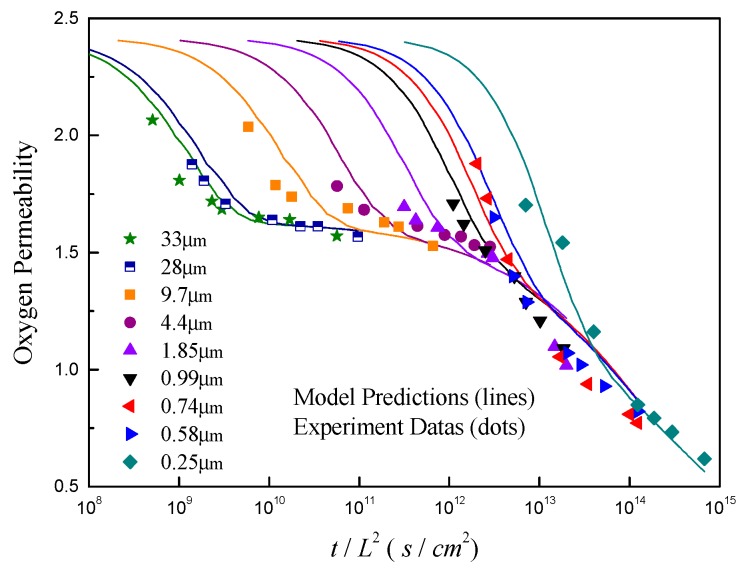
Model validation with experimental aging data for BPA–BnzDCA polymeric films with various thicknesses from 250 nm to 33 µm [21].

Previously, extensive experimental efforts have been made to reveal the aging behavior of gas separation membranes. Among these works, McCaig and Paul et al. [20,21] have shown that the experimental oxygen permeability data for thin BPA–BnzDCA films (0.25–33 μm) are remarkable. They have proved that the free volume reduction associated with physical aging behind these data occurs by two distinct simultaneous mechanisms, i.e., one is thickness-dependent, which is expected for a vacancy diffusion process, while the other is independent of thickness which is hard to describe by the diffusion mechanism. Here, from the perspective of model development, we use these published experimental data to examine our new proposed model. The permeability based on the concentration profiles are calculated and compared with the experimental data, as depicted in Figure 1. In the calculation, the pressures at upstream, x=0, and downstream, x=L, are considered as constants which provide a driving force for the transportation of gas. Due to the non-equilibrium nature of glassy polymers, the corresponding boundary conditions for the gas concentration are therefore determined by a combination of Henry’s law and the hole filling Langmuir model according to the Ref. [34], i.e., $C=\left[S_{H}+bS_{L}/(1+bp)\right]p$, where $S_{H}$ is the Henry’s law constant, $S_{L}$ and b are the Langmuir capacity and affinity constants, and p is the gas pressure at the surface. As a result of lacking experiment data, an equivalent solubility, $S^{\prime }=S_{H}\left[1+bS_{L}/\left(1+bp\right)S_{H}\right]$ of 0.367 $cm_{STP}^{3}/cm^{3}\cdot atm$ is used, which implies that $bS_{L}/\left(1+bp\right)S_{H}\approx 0.04$ based on the work of McCaig et al. [20]. The pressure for the upstream is set as 2 atm at the inlet surface, and is considered as 0 atm at outlet surface according to the experimental condition in Ref. [21]. Concerning the boundary condition for the free volume, we adopt a generalized form of $v_{f}=v_{f}^{LC}-v_{f_{r}}+v_{f_{e}}$ in this research. It means that the surface free volume may deviate from its equilibrium value of $v_{f_{e}}$ due to the impact of lattice contraction departure from its reference $v_{f_{r}}$. Supposing that γ in Equation (14) is zero, and the lattice contraction process begins at a free volume of $v_{f_{i}}$ at initial aging time (t=0) and approaches its “bulk” or glassy value of $v_{f_{g}}$, i.e., $v_{f_{r}}=v_{f_{g}}$, this boundary condition will degenerate to that used in McCaig’s model in Ref. [21], namely $v_{f}=v_{f_{e}}+(v_{f_{i}}-v_{f_{g}})e^{-t/\tau }$. Coupling factors $A_{12}$ and $A_{21}$ in the calculation are set as zero in accordance with the experiment method, in which the permeation measurements are short compared to the aging time, and the diffusion of gas on the variation of aging is negligible. The underlying coupling between mass transport and physical aging will be discussed later. The simulation parameters adapted for model validation are summarized in Table 1. All experimental data were read carefully from the reference with the help of GetData Graph Digitizer^®^.

From Figure 1, it can be seen that the predicted values are in good agreement with experimental data for both thick and thin films, which validates the accuracy of the current model. It should be noted that the empirical parameters *A* and *B*, which are needed among other models to fit the relationship between free volume and permeability with the expression of $P=Ae^{-B/v_{f}}$, are unnecessary in our calculations. All parameters can be estimated from the experiments, such as the experimental PVT relationship, and gas adsorption and diffusion data [21]. According to Equation (3), the total free volume across the film consists of diffusion and contraction components. Among these components, the diffusion part is path-dependent and thus is a function of the coordinates along film thickness, while the contraction piece is the path-independent and is only a function of aging time, as shown in Equation (14). Therefore, without considering the impact of free volume gradients ($A_{12}=0$), the decrease in the permeability of thick films, such as *L* ≥ 9.7 µm, is caused mainly by the accelerated degradation of gas flux induced by free volume contraction, while the reductions in the permeability of thin films are attributed to the loss of gas flux because of the diffusion of free volume toward the membranes surface. These findings are consistent with the dual-mechanism results in the McCaig’s model. 

**Figure 2:**
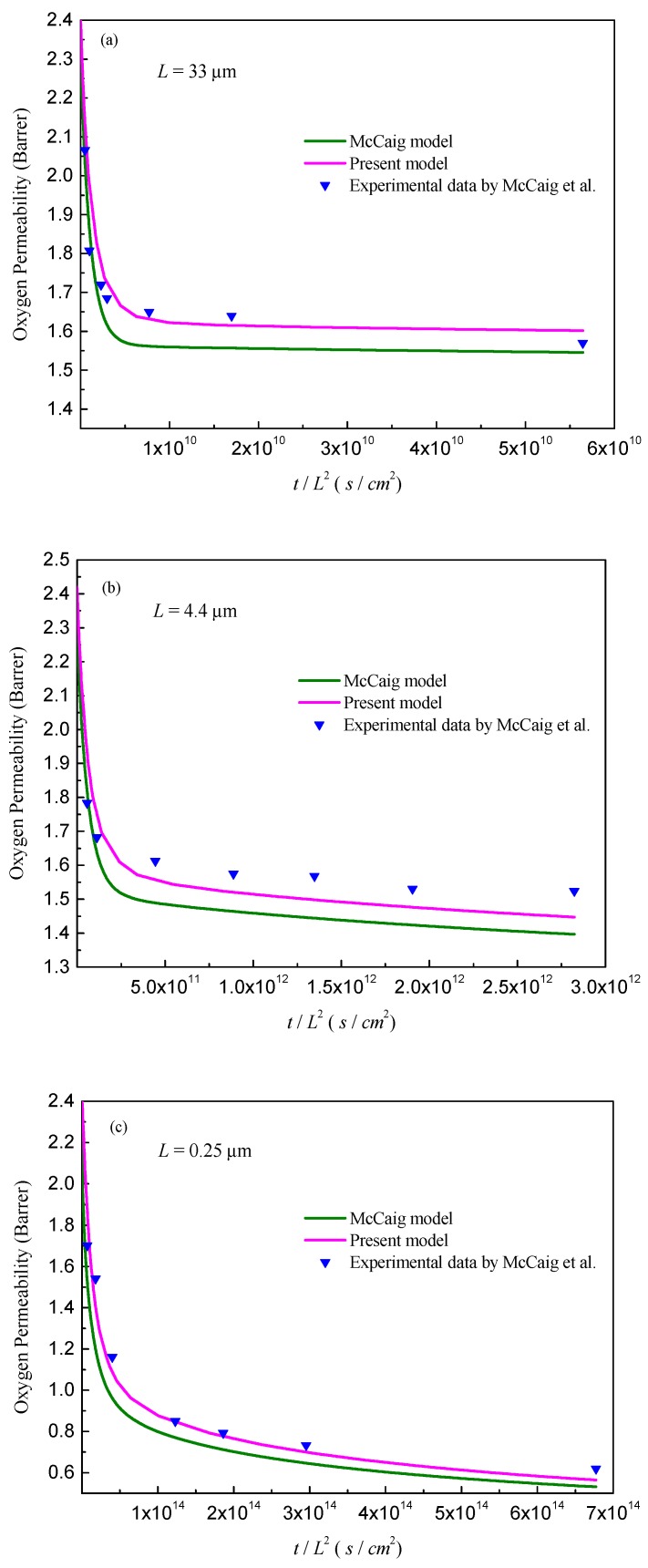
Comparison of the present model with the existing McCaig’s model [21]: (**a**) Film thickness: *L* = 33 μm; (**b**) *L* = 4.4 μm; and (**c**) *L* = 0.25 μm.

To further check the accuracy of predictions, the MC model is selected as a reference to compare the current concentration-based model with the results shown in Figure 2. For clarity, three typical films with thick, moderate, and thin thicknesses are selected and compared, as shown in Figure 2a–c.

It can be seen from Figure 2 that the current model is able to produce good consistency with the experimental results for these typical films. To quantify the accuracy of the predicted permeability for various film thicknesses, the values of mean relative percentage error (MRPE) are also calculated. The MRPE is defined by $\mathrm{MRPE}=\frac{1}{n}\sum_{i=1}^{n}\left|\left(p_{i}^{\mathrm{pre}}-p_{i}^{\exp}\right)/p_{i}^{\exp}\right|$, where *n* is the number of experimental data, and $p^{\mathrm{pre}}$ and $p^{\exp}$ are the corresponding predicted and experimental permeability, respectively. Through calculations, a small MRPE of −3% < MRPE < 8.5% has been obtained with the current model over a wide range of film thicknesses from 0.25 µm to 33 µm, which reconfirms the accuracy of the developed mathematical model. Comparing with the MC model, in which the permeability over the film thickness depends only on the free volume profile, the permeability in the current model relates further to the mass concentration gradient distribution as shown in Equation (23). Thus, the modified consistency in permeability depicted in Figure 2 can be attributed to the consequences of the combination of concentration gradient and free volume.

### 4.2. Coupling between Mass Transport and Physical Aging

**Figure 3:**
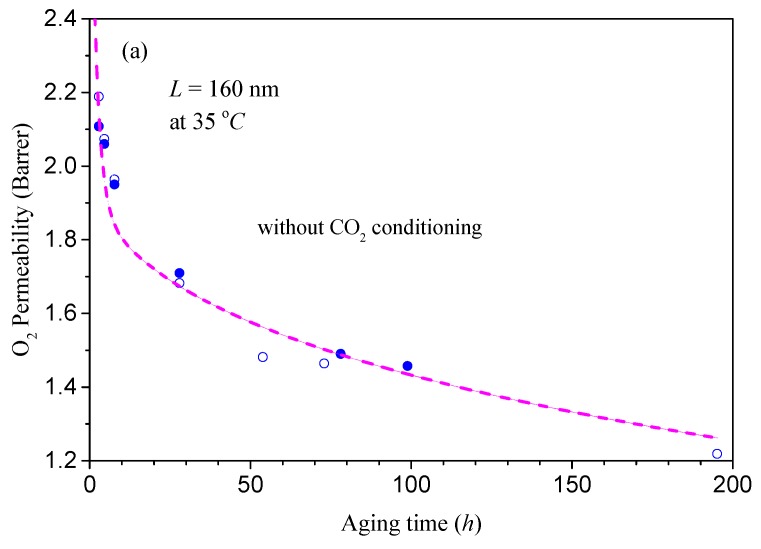

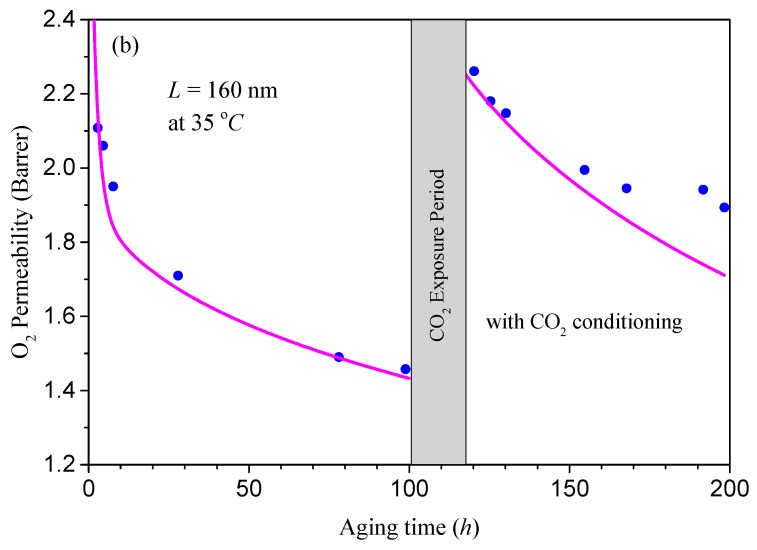
Comparison of the experimental data (symbols) [29] with the model predictions (lines): (**a**) Without CO_2_ exposure; (**b**) with short-time CO_2_ exposure.

**Figure 4:**
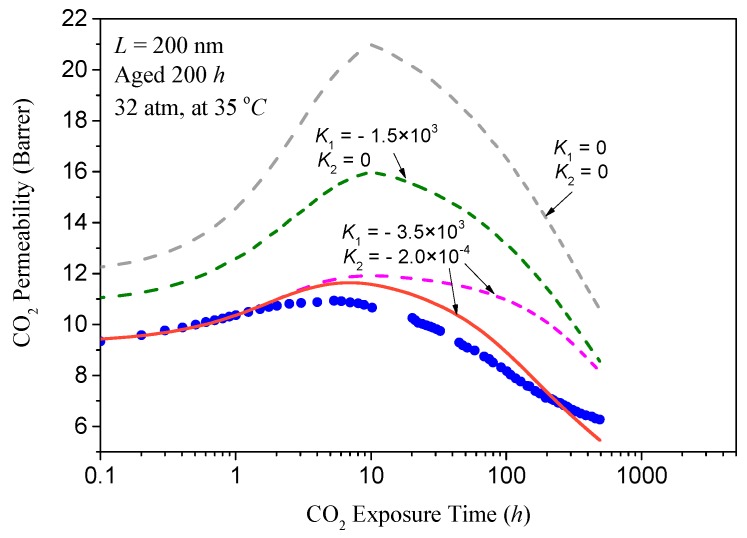
Comparison of the experimental long time CO_2_ permeability data (symbols) with the coupled model predictions (lines). Dash lines are the results based on the assumed renewal process of lattice contraction stated in step 2 of this section. The solid line is the result of coupling and accelerated diffusion with parameters of $k_{1}=3.5\times 10^{3}$,$k_{2}=-2\times 10^{-4}$, and $A_{22}=4.72\times 10^{-11}cm^{2}/s$.

One of the most important features in the present model is that the coupling between mass transport and physical aging has been taken into account. In order to show the potential coupling effect, here we consider the experimental permeability data reported by Horn and Paul [29]. To organize the analysis, the simulations are conducted in three steps: 1. Experimental permeability data of O_2_ in Matrimid^®^ without CO_2_ conditioning is used to fit the pending parameters within the theoretical model shown in Section 3; 2. Horn and Paul have shown that the permeability of O_2_ in Matrimid^®^ in both thick and thin films increases considerably after CO_2_ exposure. To mimic this phenomenon, we assume that the free volume induced by lattice contraction $v_{f}^{LC}$ may follow a reactivation process, i.e., $v_{f}^{LC}$ tends to restore its initial values, $v_{f_{i}}$, during the CO_2_ exposure process. Hence, this assumption is examined by the permeability data of O_2_ in Matrimid^®^ with CO_2_ exposure; 3. With the help of the parameters and mechanism obtained in the first two steps, we then simulate CO_2_ permeation behavior for long exposure times to check the underlying coupling between mass transport and physical aging. These results are depicted in Figure 3a,b, and Figure 4. The parameters adopted for simulation are listed in Table 2.

Figure 3a shows the comparison of the experimental permeability data of O_2_ without CO_2_ exposure with the model calculations described in Section 3. In Ref. [29], the permeability of O_2_ was tracked for two thin films with the same thickness of 160 nm. One of the films did not experience any CO_2_ exposure and the permeability of O_2_ was tracked until the film had aged 200 h at 35 °C (the results are expressed with hollow circles in Figure 3a), while the other was exposed to CO_2_ after the same aging for 100 h (the experimental data are shown with solid circles in Figure 3a,b). In the simulation, all these data are utilized to improve the fitting accuracy on the permeability of O_2_. The obtained imitated parameters, such as $B_{f}$, τ, and $A_{22}$, are listed in Table 2. From Figure 3a, it can be seen that these parameters can provide an accurate description of the entire experimental behavior. The calculated value of mean relative percentage error (MRPE) is about 3.2%.

With the help of the obtained parameters discussed above, we perform further simulations to describe the permeability data of O_2_ in Matrimid^®^ with CO_2_ exposure with the results depicted in Figure 3b. In the simulation, as the permeability of oxygen rises rapidly after the short-time exposure of CO_2_ (less than 20 h) according to the experimental results shown with solid circles in Figure 3b, we consider that the intrinsic change of free volume is dominated by lattice variation $v_{f}^{LC}$, since the diffusion of free volume is comparatively much slower. Due to the swelling of carbon dioxide, the free volume induced by lattice contraction $v_{f}^{LC}$ is assumed to be renewed during the CO_2_ exposure stage. Hence, the regenerative time, $\tau_{R}$, within CO_2_ exposure as well as the subsequent relaxation time, $\tau_{s}$. after the disturbance of CO_2_ deservedly need to be identified at this step. Through numerical analysis, the optimized values for $\tau_{R}$ and $\tau_{s}$ are about 2.5 h and 2000 h, respectively. From Figure 3b, it can be observed that the new modified model with the assumption on the regeneration of lattice contraction is able to produce the experimental results well. The value of mean relative percentage error (MRPE) with these parameters is about 3.6%. Previously, Paul et al. [29,36] have suggested that the increase in permeability of glassy films after CO_2_ exposure can be ascribed to the enhanced molecular motions induced by free volume increments, and the polymeric film may not immediately return to its previous state once the plasticizer CO_2_ is removed. Our findings here provide some details for their opinions, with the recommendation that the increase in free volume can be described by the regeneration of lattice contraction. Moreover, the values of $\tau_{R}=2.5\;\mathrm{hr}$ and $\tau_{s}=2000\;\mathrm{hr}$ imply that this regeneration process due to plastication can be finished within a few hours, while the following recovering time after the plasticizer is removed may continue for a few months. This result implies that the reduction of the disturbed free volume after CO_2_ removal may interact with the subsequent mass transport. For this reason, the discrepancy between the simulation results and the experimental values after CO_2_ removal may be caused by the fact that the subsequent transports of free volume and gas molecules are coupled, which will be elucidated further in the following sections.

Based on the above results, Figure 4 shows the comparison between the model calculations (lines) stated in step 3 and the experimental long-time CO_2_ exposure data (symbols). When the coupling factors $A_{12}$ and $A_{21}$ are zeros, i.e., $k_{1}=A_{12}/A_{11}=0$ and $k_{2}=A_{21}/A_{22}=0$, the transport of gas molecules and the aging process are independent. From Figure 4, it can be found that the model agrees only with the shape and the peak position of the experimental aging data, indicating that the assumed renewal process of lattice contraction as well as the obtained regenerative time, $\tau_{R}$, in step 2 are appropriate. As $A_{12}$ and $A_{21}$ rise, the transport of gas molecules and the aging process become more closely coupled, leading to a significant decrease of the flux of the gas, and thus the values of the predicted response begin to resemble the experimental data. After a set of simulations, it can be observed from Figure 4 that the enhanced reproduction of the experimental values is mainly attributed to the increase in the coupling factor $k_{1}$, suggesting that the impact of the free volume gradient on the mass concentration may be significant. The relatively optimized values for $k_{1}$ and $k_{2}$ are about −3.5 × 10^3^ and −2 × 10^−4^, respectively. Due to the lack of experimental data and related mechanism-based explanation, we assume that only the renewal process of lattice contraction occurs when the film experiences the CO_2_ exposure. Since the corresponding regenerative time $\tau_{R}$ obtained in step 2 is about 2.5 h, it is expected to find in Figure 4 that the model reproduces the permeability data very well during the rejuvenation stage. The failure of the model mentioned above to represent the subsequent experimental data indicates that other processes, such as re-relaxation or acceleration of diffusion, may also occur after the lattice contraction recovery process. With these amendments, we believe that the predicted values for the long-time CO_2_ permeability will be further optimized. For example, the addition of an accelerated diffusion after the renewal process of lattice contraction will significantly increase the accuracy of the forecast, which has already been depicted with a magenta solid line in Figure 4. Of course, these conjectures require further experimental evidence in future works.

**Figure 5:**
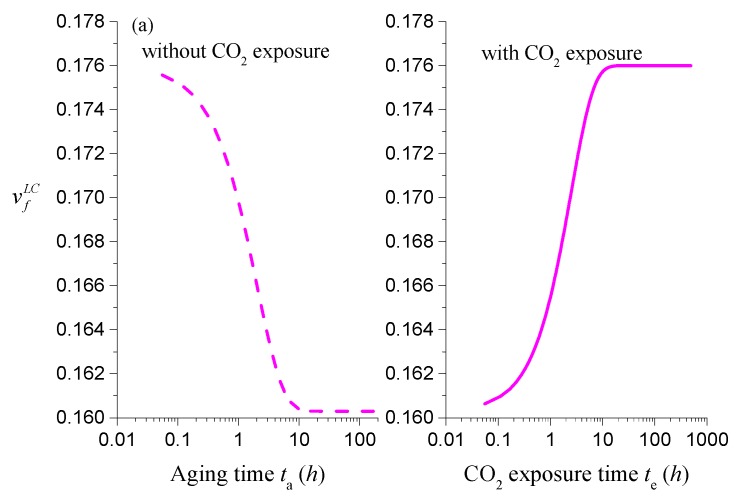

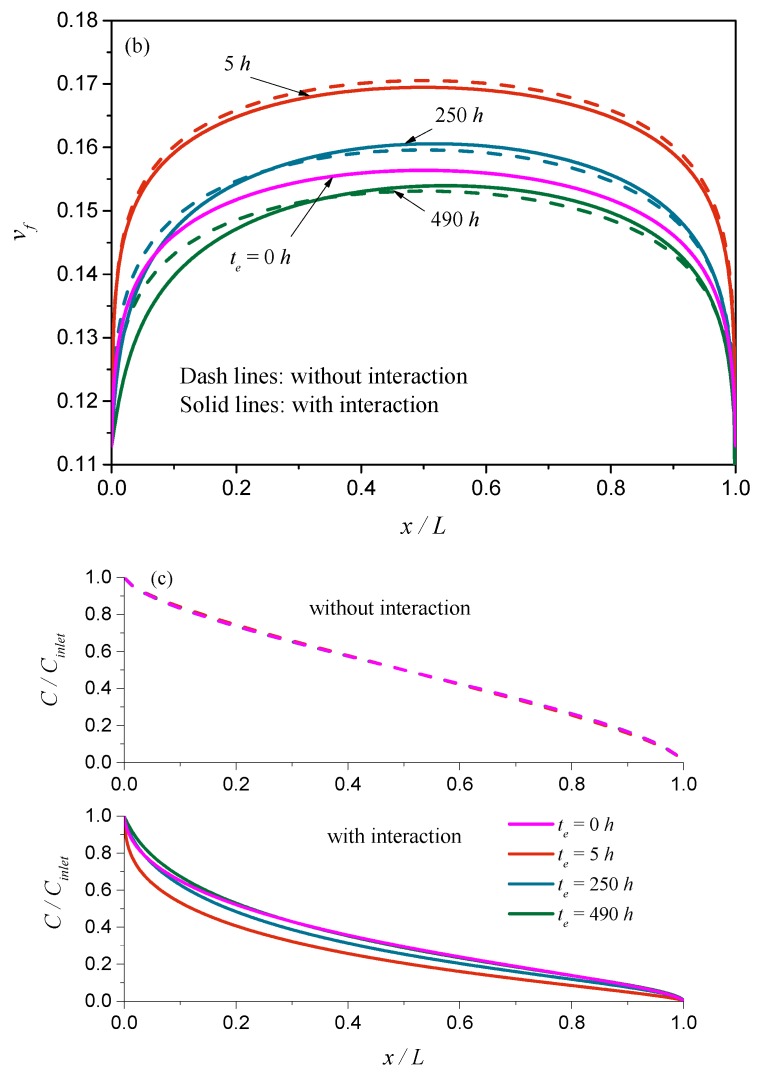
Simulated profiles of the lattice contraction part (**a**) and the free volume (**b**), as well as the gas concentration (**c**) across film thickness upon different aging times.

In order to further unveil the coupling mechanism, it is useful to illustrate how the free volume and mass concentration vary with different values of the CO_2_ exposure time $t_{e}$. The corresponding results are developed and depicted in Figure 5a–c. In Figure 5a, we observe that the lattice contraction part $v_{f}^{LC}$ decreases with time at the previous aging stage, and recovers quickly after CO_2_ exposure due to plasticization. For this reason, it can be seen in Figure 5b that the total free volume is enhanced at the initial exposure time. As $v_{f}^{LC}$ approaches to its equilibrium after long-time exposure, the following elimination of free volume is therefore attributed to the free volume diffusion. These findings are, as expected, consistent with the ideas discussed in previous sections.

Moreover, it is important to find in Figure 5c that the concentration profile remains unchanged if the coupling between mass transport and aging is neglected, indicating that the concentration gradient does not change with the elapse of aging time. Hence, if the concentration gradient is approximately set as 1, i.e., $\partial \bar{C}/\partial \bar{x}=1$, according to the calculated results, the corresponding permeability as shown in Equation (23) can then be simplified as:

(24) $$P\left(t\right)=\frac{1}{\Delta p}\int_{0}^{L}A_{11}e^{-B_{c}/v_{f}}\frac{\partial C}{\partial x}dx=A_{11}\frac{C_{inlet}}{\Delta p}\int_{0}^{1}e^{-B_{c}/v_{f}}\frac{\partial \bar{C}}{\partial \bar{x}}d\bar{x}\approx \int_{0}^{1}A^{\prime }e^{-B_{c}/v_{f}}d\bar{x}$$

where $A^{\prime }=A_{11}C_{inlet}/\Delta p$ is constant. This result shows that the current coupling model can degenerate into the classical free volume-based model, in which $P=Ae^{-B/v_{f}}$ and $B=B_{c}$. This result further validates that the improved consistency in permeability, as discussed in Section 4.1, is mainly ascribed to the introduction of concentration gradients. In addition, it is remarkable to find in Figure 5c that the concentration profile varies significantly when the interaction between mass transport and aging is considered. Since the concentration at the inlet surface ($\bar{x}=0$) is much higher than that at the outlet side ($\bar{x}=1$), the free volume and concentration gradients are therefore changed significantly at the inlet side, resulting in a local strong coupling between mass transport and physical aging.

## 5. Conclusions

In this paper, we develop a continuum model to reveal the underlying coupling between mass transport and physical aging. In order to validate the model and apply it to demonstrate the coupling phenomenon, the obtained governing equations are embodied and employed to simulate the transport behavior of gas within aging polymeric membranes. Since the flux of gas concentration is analyzed, the permeability is thus expressed as an integral function of the concentration gradient, free volume, and free volume gradient, which differs from the existing free volume-based method. By comparing the predicted values of the permeability of O_2_ in BPA–BnzDCA films with the experimental data as well as the free volume-based dual-mechanism results, we show that due to the introduction of the concentration gradient, the current model is able to produce relatively better consistency with the experimental data for films with thicknesses from 0.25 to 33 µm. More importantly, to mimic the increase in permeability of O_2_ within Matrimid^®^ thin films after short-time exposure to CO_2_, we proposed at the first time that the free volume induced by lattice contraction may be renewed during the CO_2_ exposure stage. With the help of this assumption, our model is therefore capable of producing the experimental permeability behavior of O_2_ after short-time CO_2_ exposure well. In addition, by applying the validated straightforward permeability calculation method and free volume recovery mechanism, we also have carried a set of simulations on the experimental permeability of CO_2_ with the results, suggesting that the transports of CO_2_ and free volume are coupled. Due to the strong coupling, the free volume and concentration gradients change significantly, especially at the high concentration inlet side.

Remarkably, polymer membranes with proper fillers, especially nanoparticle or metal-organic frameworks, are being proved as effective ways to enhance the film performance in water treatment [37,38], gas separation [39,40], and organic solvent nanofiltration [41,42,43]. Although the above model has revealed the underlying aging mechanism of pure polymers with transported mass, it is a wonder that the aging process of polymer composites with inorganic fillers can be depicted by the present equations. According to the continuum thermodynamics, we may infer that the general model given in Section 2 can be applied to micro-particle filled polymers. Unfortunately, it is difficult to directly describe the transport properties of aging polymer films containing nano-fillers, owing that the nanoparticles have tremendous effects on the evolution of free volume in glassy nanocomposites [44]. Nevertheless, it would be a potential solution since due to the increase in specific surface area of the filler or the interfacial area-per-unit volume between the polymer and the nanoparticle, the extra interfacial energy should be taken into account in the Gibbs free energy for updating the present method in the future. In addition, by introducing proper additional parameters, such as stress and other internal state variables which can refer to the published references [14] and [30], the more generalized model can be obtained via a similar derivation process and even applied to describe the viscoelastic behavior of these films subjected to the stress. It is noticed that the developed methodology is based on the work of Weitsman [14] who considered the aging response with moisture transport. Thus, the existing equations in Section 2 are also theoretically applicable to describe the aging performance of polymeric glass in vapor environments. For this application, how to specify the aging parameters would be critical, as the glass transition temperature is primarily influenced by the moisture concentration [45]. 

## Figures and Tables

**Table 1:** Model parameters for bisphenol-A benzophenone-dicarboxylic acid (BPA-BnzDCA) films.

Parameters	Reference
$v_{f_{i}}=0.160$, $v_{f_{g}}=0.149$, $v_{f_{e}}=0.102$, $B_{f}=3$, $B_{c}=0.839$, τ=3.5h, γ=0,	Ref. [21]
^a^$A_{22}=2.49\times 10^{-6}cm^{2}/s$, ^b^$A_{11}=9.48\times 10^{-6}cm^{2}/s$	Estimated

^a^. $A_{22}$ is estimated by $A_{22}=D_{f}e^{B_{f}/v_{f_{g}}}$, where $D_{f}=4.48\times 10^{-15}cm^{2}/s$, as obtained from Ref. [21]; ^b^. $A_{11}$ is estimated by $A_{11}=D_{c}e^{B_{c}/v_{f_{g}}}$, where $D_{c}=3.4\times 10^{-8}cm^{2}/s$, according to Ref. [20].

**Table 2:** Model parameters for Matrimid^®^ thin films.

Procedures	Parameters	Reference
Step 1	$v_{f_{i}}=0.176$, $v_{f_{e}}=0.1131$, $B_{c}=0.839$,	Refs. [11,24]
	^a^$v_{f_{g}}=0.1603$,	Refs. [4,11]
	^b^$A_{11}=8.74\times 10^{-7}cm^{2}/s$, ^c^$C_{\mathrm{inlet}}=13.83cm_{STP}^{3}/cm^{3}$	Refs. [4,35]
	$B_{f}=2.0$, τ=2.0 hr, $A_{22}=1.18\times 10^{-11}cm^{2}/s$	Fitted
Step 2	$\tau_{R}=2.5\;\mathrm{hr}$, $\tau_{s}=2000\;\mathrm{hr}$	Fitted
Step 3	^c^$C_{\mathrm{inlet}}=37cm_{STP}^{3}/cm^{3}$, $B_{c}=0.860$	Ref. [11]
	$k_{1}=A_{12}/A_{11}=-3.5\times 10^{3}$, $k_{2}=A_{21}/A_{22}=-2.0\times 10^{-4}$	Fitted

^a^. $v_{f_{g}}$ is estimated by $P_{g}=Ae^{-B_{c}/v_{f_{g}}}$, where $P_{g}=2.12\;\mathrm{Barrer}$, A=397, and $B_{c}=0.839$ can be obtained from Refs. [4,11]. ^b^. $A_{11}$ is obtained from $A_{11}=D_{c}e^{B_{c}/v_{f_{g}}}$, where $D_{c}=4.66\times 10^{-9}cm^{2}/s$, according to Ref. [35]. ^c^. $C_{inlet}$ is estimated by Henry’s law, i.e., $C_{inlet}=S\times p$, where p is the surface gas pressure and S is the solubility which can be obtained by $S=P_{g}/D_{c}$.

## List of Symbols and Abbreviations

*A*,*B*	material constants in Paul’s equation	**q**	flux of heat
*A*_*ij*_	coefficients in current coupling model	*R*	thermodynamic potential
*C*	mass concentration	*S*	solubility
**f**	flux of gas	*s*	entropy densities of the mass mixture
*G*	Gibbs free energy	*T*	temperature
*h*	enthalpy	*u*	internal energy density
*k*	Boltzmann’s constant	*v*_f_	fractional free volume
*L*	membrane thickness	***x***	thermodynamic force vectors
*L_ij_*	phenomenological coefficients	γ	material constant
*m*_0_	mass of a single molecular	μ	chemical potential
*N*_*c*_	flux of penetrant mass	ρ	mass density
*P*	permeability	τ	relaxation time
*p*	pressure	Ψ	flux of free volume
